# Case report: Endovascular coil embolization of an aneurysm at the origin of the accessory middle cerebral artery from the A1 segment as the collateral artery to twigs

**DOI:** 10.3389/fneur.2023.1078173

**Published:** 2023-04-20

**Authors:** Lei Zhang, Han Su, Jinlu Yu

**Affiliations:** ^1^Department of Neurosurgery, Daqing Oilfield General Hospital, Daqing, China; ^2^Department of Neurosurgery, First Hospital of Jilin University, Changchun, China

**Keywords:** twig-like middle cerebral artery, accessory middle cerebral artery, anterior cerebral artery, aneurysm, embolization

## Abstract

An aneurysm at the origin of the accessory middle cerebral artery (AccMCA) from the A1 segment of the anterior cerebral artery (ACA) as the supplying artery of a twig-like MCA is exceptional. In this study, we reported on such a case and presented a review of the relevant literature. A 56-year-old male suffered a subarachnoid hemorrhage. Digital subtraction angiography confirmed a twig-like MCA and a ruptured aneurysm at the origin of the AccMCA. Endovascular coil embolization of the aneurysm was performed. After the microcatheter was positioned in the aneurysm, soft coils were delivered to complete the embolization. Postoperatively, the patient recovered uneventfully. One month later, the patient returned to his job without any neurological deficits. Postoperative computed tomography at the 3-month follow-up showed that the brain tissue was normal. By reporting our case and reviewing the relevant literature, we found that endovascular coil embolization for such aneurysms at the AccMCA origin is feasible in certain cases.

## Introduction

A twig-like middle cerebral artery (MCA) or MCA twig is an uncommon lesion in which a plexiform network of small vessels replaces the M1 segment. The embryological interruption of MCA trunk genesis may be the cause of these lesions (1, 2). Twig-like MCAs can cause a hemorrhagic or ischemic event (3, 4). A twig-like MCA has a complex angioarchitecture, and the accessory MCA (AccMCA) from the A1 segment of the anterior cerebral artery (ACA) can serve as an important collateral artery for MCA twigs (5, 6). In cases of twig-like MCAs, due to hemodynamic stress, aneurysms can occur at the origin of the AccMCA from the ACA (5–11).

Treatment is mandatory for ruptured aneurysms at the origin of the AccMCA; however, clipping may carry a risk of neurological complications because of the deep location and destruction of the collateral circulation (12). The International Subarachnoid Aneurysm Trial (ISAT) and its follow-up study confirmed the effect of endovascular coil embolization for ruptured aneurysms (13, 14). Therefore, endovascular coil embolization can be applied for aneurysms at the origin of the AccMCA (15).

However, endovascular coiling for an aneurysm at the origin of the AccMCA as the supplying artery of a twig-like MCA is exceptional. In this study, we reported on the application of endovascular coiling for such an aneurysm and reviewed the relevant literature.

## Case report

**Figure 1:**
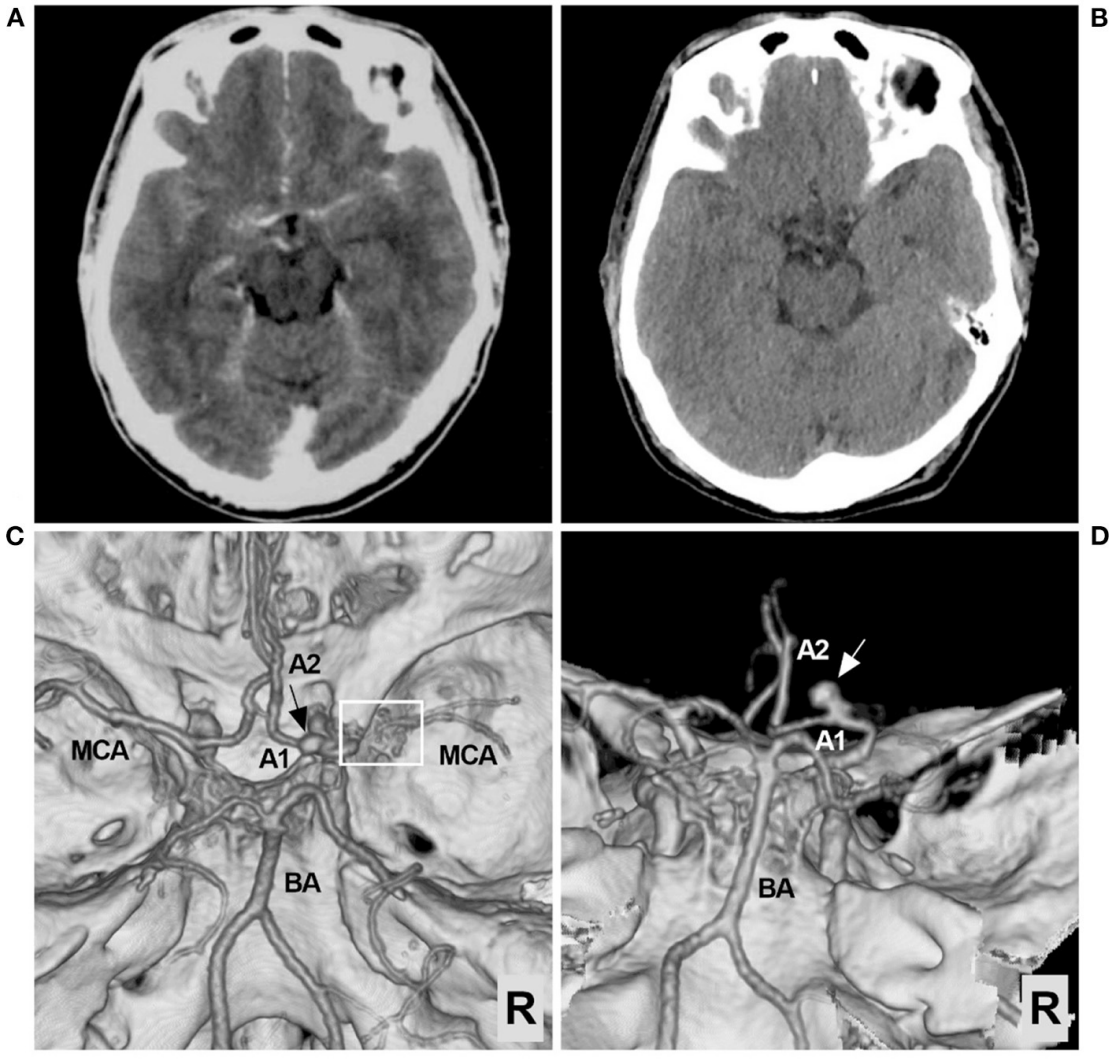
Preoperative CT and CTA images. **(A)** CT showing the extensive SAH, focusing on the left suprasellar cistern and the base of the Sylvian fissure. **(B)** Six days later, repeat CT showing absorption of the SAH. **(C)** CTA of the superior-inferior view showing the arterial network of the right proximal MCA (frame) and an aneurysm (arrow). **(D)** CTA of the posterior-anterior view showing the aneurysm in detail; the aneurysm (arrow) was located in the A1 segment of the ACA. ACA, anterior cerebral artery; A1, first segment of the ACA; A2, second segment of the ACA; BA, basilar artery; CT, computed tomography; CTA, CT angiography; MCA, middle cerebral artery; R, right; SAH, subarachnoid hemorrhage.

A 56-year-old male with an unremarkable medical history presented with an acute onset of headache. The patient was of Chinese Han nationality and had no history of drug abuse or surgical treatment of craniocerebral diseases. The computed tomography (CT) obtained at the local hospital showed a grade 1 subarachnoid hemorrhage (SAH) on the modified Fisher scale (Figure 1A). After 6 days of conservative treatment, the patient was admitted to our hospital. CT was repeated and showed that the SAH had been absorbed (Figure 1B). CT angiography showed the arterial network of the right proximal MCA and an aneurysm on the A1 segment of the ACA (Figures 1C, D). The patient could correctly obey commands during the physical examination, and his condition was classified as grade I on the Hunt-Hess scale. The limb muscle strength was grade V, and the Babinski sign was positive in both lower limbs.

**Figure 2:**
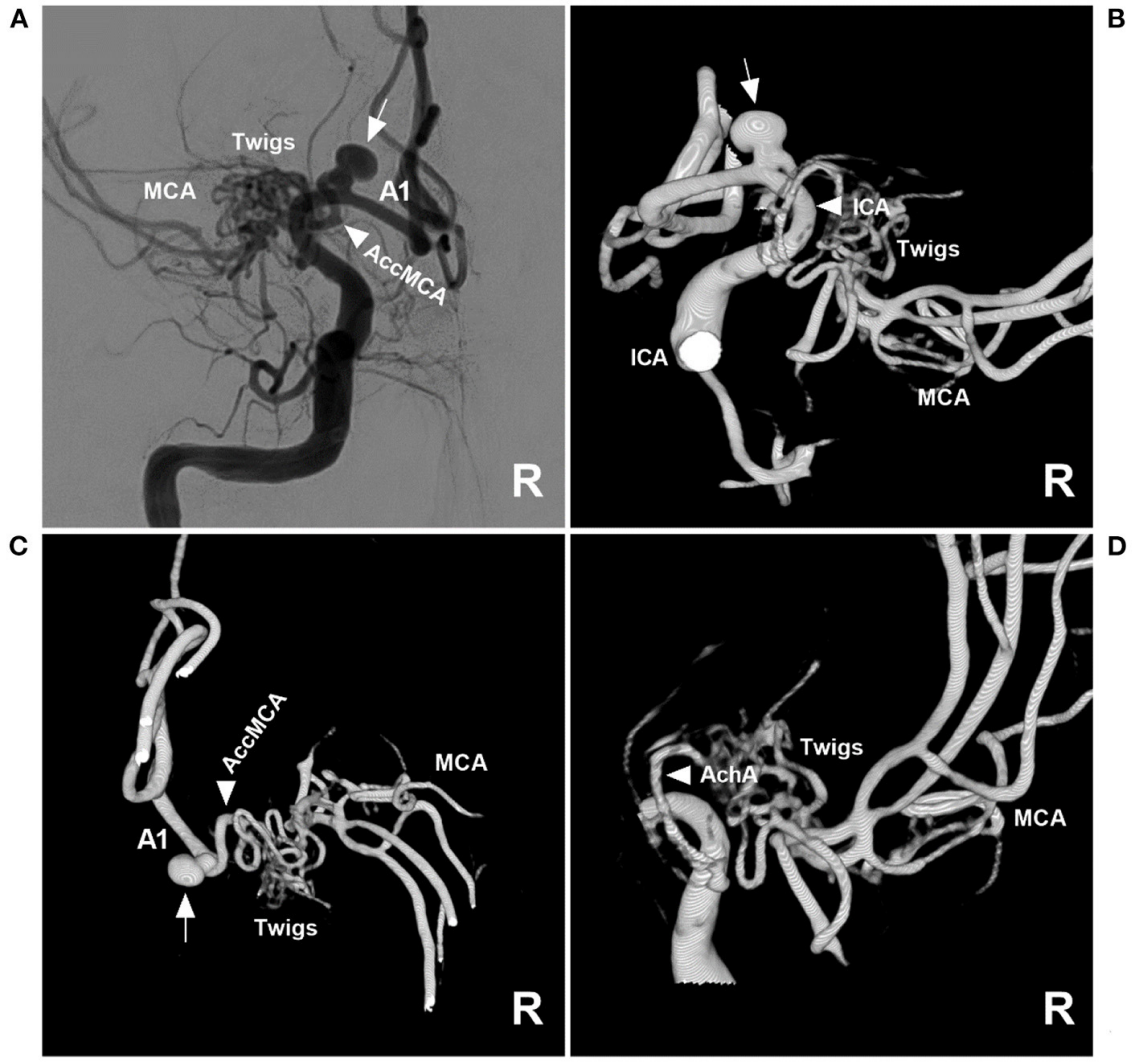
Angioarchitecture of twig-like MCA. **(A)** Two-dimensional DSA of the right ICA showing a right twig-like MCA and an aneurysm (arrow) at the origin of the AccMCA from the A1 segment. **(B)** Three-dimensional DSA showing that the ICA (arrowhead) did not give off the branch supplying the twig-like MCA; the arrow indicates the aneurysm. **(C)** Three-dimensional DSA showing the AccMCA (arrowhead) as a collateral artery from the A1 segment supplying the twig-like MCA; the arrow indicates the aneurysm. **(D)** Three-dimensional DSA showing that the AchA (arrowhead) supplies the twig-like MCA. A1, first segment; AccMCA, accessory middle cerebral artery; ACA, anterior cerebral artery; AchA, anterior choroidal artery; DSA, digital subtraction angiography; ICA, internal carotid artery; MCA, middle cerebral artery.

**Figure 3:**
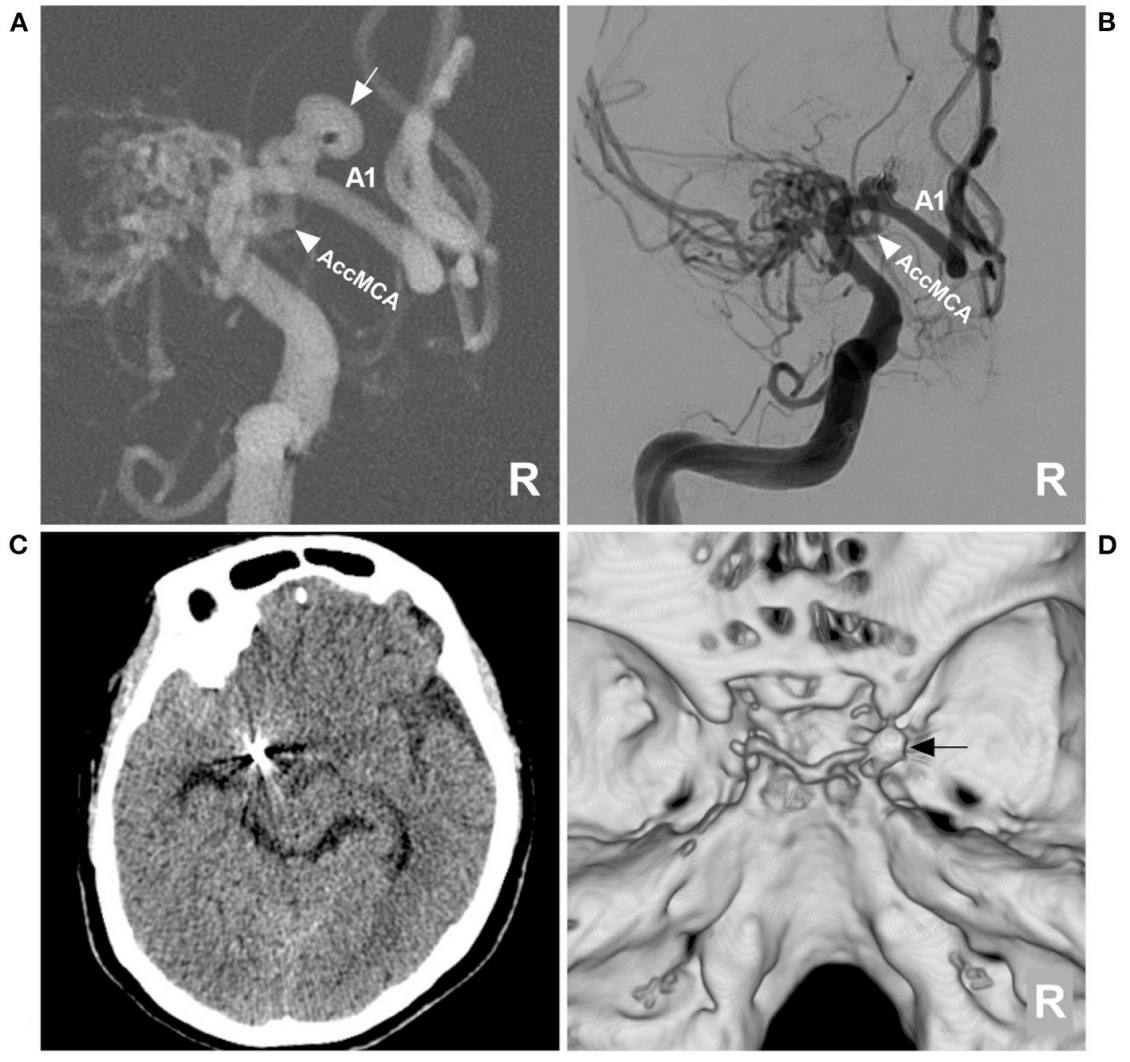
Aneurysm coiling and follow-up CT. **(A)** DSA roadmap navigation of the right ICA showing that the microcatheter was positioned into the aneurysm sac (arrow); the arrowhead indicates the AccMCA. **(B)** Postoperative DSA of the right ICA showing that the aneurysm was completely coiled and that the AccMCA (arrowhead) was preserved. **(C)** Follow-up CT showing normal brain tissue. **(D)** CT reconstruction showing the coils (arrow). A1, first segment; AccMCA, accessory middle cerebral artery; CT, computed tomography; DSA, digital subtraction angiography; ICA, internal carotid artery; MCA, middle cerebral artery; R, right.

Then, endovascular coil embolization for the aneurysm was planned. During the treatment, digital subtraction angiography (DSA) confirmed that the right MCA was twig-like, an aneurysm with a diameter of 4 mm was located at the origin of the AccMCA from the A1 segment of the ACA, and the AccMCA was a collateral artery for the MCA twig (Figure 2). After the three-dimensional reconstruction of the DSA data, the best projection degree showed the aneurysm sac and its neck. An Echemon-10 microcatheter (Medtronic, Irvine, CA, USA) was used to perform the coiling [Axium Prime coils: 3.5 mm × 10 cm, 2 mm × 6 cm, 1.5 mm × 3 cm (Medtronic, Irvine, CA, USA)], and the aneurysm was completely embolized (Figures 3A, B).

Postoperatively, the patient recovered uneventfully. One month later, the patient returned to his job without any neurological deficits. A postoperative CT at the 3-month follow-up showed that the brain tissue was normal (Figures 3C, D).

## Discussion

**Figure 4:**
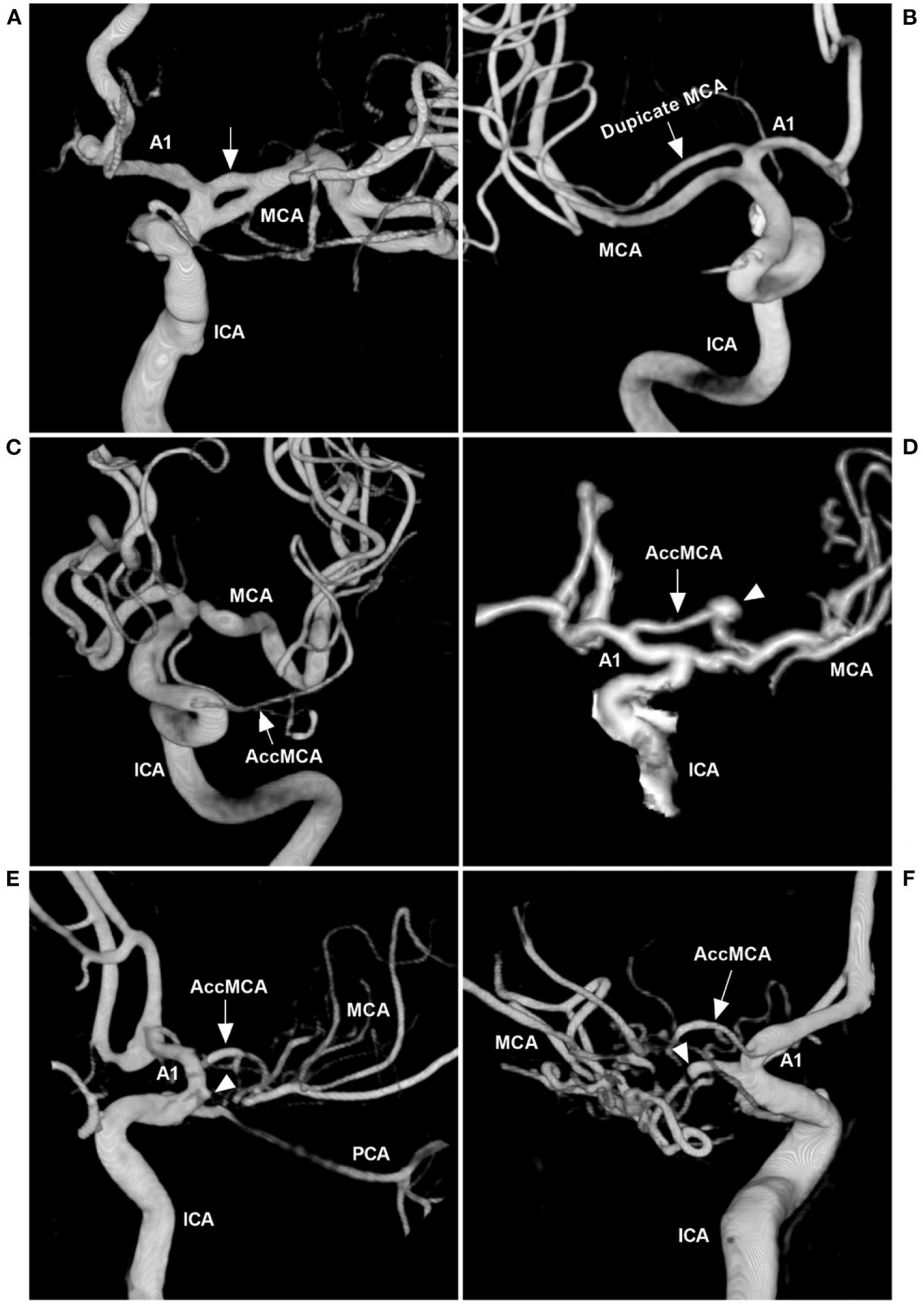
MCA anomalies on angiography. **(A)** Three-dimensional DSA of the ICA showing a fenestration (arrow) at the beginning of the MCA. **(B)** Three-dimensional DSA showing a duplicate MCA (arrow). **(C)** Three-dimensional DSA showing a type 1 AccMCA (arrow) from the ICA trunk. **(D)** Three-dimensional DSA showing a type 2 AccMCA (arrow) with complete anastomosis between the A1 middle segment and MCA forming a large fenestration; the arrowhead indicates an aneurysm. **(E)** Three-dimensional DSA showing a twig-like MCA supplied by the AccMCA (arrow) from the A1 end; the MCA did not supply the twig-like MCA, and the MCA origin presented with a protrusion (arrowhead). **(F)** Three-dimensional DSA showing that the twig-like MCA was supplied by the AccMCA from the A1 origin (arrow); the MCA supplied the twig-like MCA (arrowhead). A1, first segment; AccMCA, accessory middle cerebral artery; CT, computed tomography; DSA, digital subtraction angiography; ICA, internal carotid artery; MCA, middle cerebral artery; PCA, posterior cerebral artery.

MCA anomalies mainly included fenestration, duplication, AccMCA, and twig-like MCA, which occur less frequently than those of other major intracranial arteries (Figure 4) (6, 16–19). An MCA fenestration is defined as segmental duplication, presenting as a vessel with two distinct endothelium-lined channels (Figure 4A) (20). A duplicate MCA is a direct bifurcation near the internal carotid artery (ICA), lacking the essential bifurcation or trifurcation at the distal end of the M1 portion (Figure 4B) (21). AccMCAs are probably residual congenital arteries that can arise from the ACA at different locations, with an angiographic incidence of 0.3–0.4% (Figures 4C, D) (22–24).

AccMCAs are essential collateral arteries that supply the MCA territory. They can be classified into three types according to the original site, as follows: the ICA trunk (type 1), the proximal A1 segment (type 2), and the distal A1 segment or junction of the anterior communicating artery itself (type 3) (25). In our case, the AccMCA arose from the proximal A1 and was therefore classified as type 2 (Figure 2). Due to the hemodynamic stress of the collateral artery, aneurysms can occur at the origin of the AccMCA (26–29). Although they have rarely been reported, we found 20 cases of aneurysms at the origin of the AccMCA in our previous review (16).

Twig-like MCAs, as a rare anomaly, have a prevalence ranging from only 0.11 to 1.17% (30) and exhibit the following radiological features on DSA: an abnormal, plexiform, multiple-channel arterial network replacing the M1 segment; a nearly normal distal MCA caliber with anterograde blood flow; permissive collateral circulation from the ACA and posterior cerebral artery; lenticulostriate arteries arising from the twigs; and the absence of transdural collaterals (1, 30). The case presented in this report confirmed the angiographic diagnostic criteria of twig-like MCAs (Figure 2).

Twig-like MCAs can be supplied by many anomalous arteries, including the steno-occlusive MCA, anomalous branches originating from the A1 and A2 segments of the ACA, the AccMCA, the anterior choroidal artery (AchA), or the ICA terminus (Figures 4E, F) (3, 30). In our case, the main suppliers included AccMCA and AchA (Figure 2). With the AccMCA as a collateral artery of the twig-like MCA, an aneurysm can occur at its origin (31). Associated aneurysms are reported in nearly 40% of cases of twig-like MCAs, suggesting hemodynamic stress and structural vulnerability, and the aneurysms can be inside or outside the twig (3, 30, 32). In our case, the aneurysm at the AccMCA origin was outside the twig.

In the Serrano-Rubio et al. review in 2022, there were 42 cases in the international literature of aneurysms associated with twig-like MCAs, most of which were ruptured (31). In this report, although cases of aneurysms at the origin of the AccMCA were collected, the review was not as extensive. Due to the rarity of aneurysms at the AccMCA origin on the A1 segment, after updating the search data, seven cases, including ours, were collected and summarized in Table 1. These cases are as follows. In 1994, Han et al. reported a first case in which a concomitant twig-like MCA was combined with an aneurysm at the AccMCA originating from the ACA (5). Later, Cekirge et al. (6), Kim et al. (7), Sakai et al. (8), Fukuda et al. (9), and Soejima et al. (10) also reported similar cases in which the associated aneurysms were clipped or coiled, with good outcomes.

**Table 1:** Clinical data from the literature review.

**No**.	**References**	**Age/sex**	**Onset**	**AccMCA origin**	**Aneurysm location**	**Aneurysm size**	**Coiling or clipping**	**Other associated anomaly**
1	Han et al. (5)	34/F	SAH	Close to A1 origin	Junction of AccMCA and A1 segment	4.5 mm	Clipping	No
2	Cekirge et al. (6)	32/M	SAH/IVH	Close to A1 origin	Junction of AccMCA and A1 segment	<5 mm	Coiling	No
3	Kim et al. (7)	64/F	SAH	Close to AcomA	Junction of AccMCA and A1 segment	<5 mm	Clipping	AcomA aneurysm
4	Sakai et al. (8)	65/F	SAH	Close to A1 origin	Junction of AccMCA and A1 segment	<5 mm	Clipping after coiling recurrence	Aneurysm in MCA twigs
5	Fukuda et al. (9)	60/F	SAH/IH	Middle A1	Junction of AccMCA and A1 segment	3.5 mm	Coiling	No
6	Soejima et al. (10)	63/F	Unruptured	Close to A1 origin	Junction of AccMCA and A1 segment	1.7 mm	Clipping and bypass	AcomA aneurysm
7	Present case	56/M	SAH	Close to A1 origin	Junction of AccMCA and A1 segment	4 mm	Coiling	No

AccMCA, accessory middle cerebral artery; AcomA, anterior communicating artery; A1, first segment of anterior cerebral artery; F, female; IH, intracerebral hemorrhage; IVH, intraventricular hemorrhage; M, male; MCA, middle cerebral artery; SAH, subarachnoid hemorrhage.

Treatment is necessary for aneurysms at the origin of the AccMCA, especially ruptured aneurysms. According to the International Subarachnoid Aneurysm Trial (ISAT) in 2005, clipping has shown superiority compared with coiling in preventing rebleeding in both the short and long term (13). However, at the 18-year follow-up of the UK cohort of the ISAT in 2015, although rebleeding was more likely after coiling than after clipping, the risk was low, and the probability of disability-free survival was significantly greater in the coiling group than in the clipping group at 10 years (14).

For aneurysms associated with twig-like MCAs, clipping may carry a risk of neurological complications because of the deep location and destruction of the collateral circulation. Therefore, considering the effect of the ISAT and its follow-up study (13, 14), when we found a saccular aneurysm of the neck that was not too wide, with a diameter of 4 mm, we determined endovascular coil embolization to be feasible and applied this strategy.

Although the endovascular coil embolization in our case was successful, we believed that the sharp upturning of the microcatheter from the ICA into the A1 aneurysm was still difficult and that the stabilization of the microcatheter was poor (15). Therefore, during coiling, soft coils, such as Axium Prime coils, are recommended to prevent the microcatheter from exiting the aneurysm too early, which would result in incomplete embolization and recurrence. For instance, in the report by Sakai et al. clipping had to be performed after coiling recurrence (8).

Therefore, in certain cases, the coiling of an aneurysm at the origin of the AccMCA as the collateral artery to a twig is feasible.

## Limitations

As a result of the economic status in rural areas in China, after repeated requests, the patient only agreed to undergo a follow-up CT and refused to undergo an angiographic examination. Therefore, follow-up aneurysm embolization data could not be obtained. However, the aneurysm was saccular, and recanalization and regrowth were uncommon after complete initial coiling.

## Data availability statement

The original contributions presented in the study are included in the article/supplementary material, further inquiries can be directed to the corresponding author.

## Ethics statement

Written informed consent was obtained from the individual(s) for the publication of any potentially identifiable images or data included in this article.

## Author contributions

JY contributed to the conception and design of the study. LZ and HS collected the data. JY and HS contributed to drafting the manuscript text and preparing the figures. LZ revised the manuscript. All authors have read and approved the final version of the manuscript.
